# Examining practice effects in repeated measurements of vibration perception thresholds on finger pulps of healthy individuals – Is it possible to improve your results over a clinically relevant test interval?

**DOI:** 10.1371/journal.pone.0226371

**Published:** 2019-12-17

**Authors:** Linnéa Ekman, Jin Persson Löfgren, Lars B. Dahlin

**Affiliations:** 1 Department of Translational Medicine—Hand Surgery, Lund University, Malmö, Sweden; 2 Department of Hand Surgery, Skåne University Hospital, Malmö, Sweden; University of Bern, SWITZERLAND

## Abstract

**Aims:**

To investigate practice effects in a test-retest situation, where vibration perception thresholds (VPT) were measured in healthy subjects using a multi-frequency test method.

**Methods:**

In eight consecutive tests, VPTs were tested in the pulps of the index and little fingers at seven frequencies (8, 16, 32, 64, 125, 250 and 500 Hz). Subjects were twenty healthy adults aged 26 to 65 years (mean 46.0 ± 11.1 years; 10 male and 10 female). The subjects were examined at six tests with intervals of one month (mean 33 ± 6; time 0 to month 5) and at two additional tests with prolonged intervals (month 12 and 18). Linear mixed model analysis was performed to investigate differences over the subsequent test occasions. To examine where potential practice effects occurred, a pairwise comparison with Bonferroni correction was made.

**Results:**

Small decreases in VPTs were found in 8 out of the 14 frequencies (index finger: 8, 16, 32, 250 and 500 Hz; little finger: 16, 250 and 500 Hz) within the test period from time 0 to month 5. In tests at 12 and 18 months, VPTs were increased compared to month 5, but lowered in comparison with time 0. Hence, minor significant decreases were found in three frequencies for the index finger (125, 250 and 500 Hz) and one frequency for the little finger (250 Hz) when examining VPTs with prolonged time intervals.

**Conclusions:**

When evaluating vibration perception thresholds in a clinically relevant time period of once or twice a year, no consideration of practice effects is necessary when interpreting the results.

## Introduction

The importance of proper functioning senses in hands cannot be underestimated as it is crucial for the highly complex motor tasks required in our everyday life. To obtain the motor control, we are dependent of the vibrotactile perception, i.e. the information from mechanoreceptors which allows us to register vibrations at different frequencies [1–3].

There are several methods available for testing vibrotactile perception and gold standard is the electrophysiological nerve conduction tests. Examination of the vibration perception threshold (VPT) is an alternative method where different equipment could be used, e.g. tuning fork (128 Hz), biothesiometer for single frequencies (50 or 100 Hz) or vibrametry at multiple frequencies. Several studies have investigated the normative values of the vibration perception threshold in healthy individuals [4–7], while others have studied VPTs in patients with diseases associated with neuropathy [5, 8–12]. There is a clear consensus that loss of vibrotactile sense, shown as a higher VPT, is useful in detecting early signs of peripheral neuropathy in patients with diabetes mellitus type 1 and 2 [13–17]. Irrelevant of preferred methods, VPT testing is low-cost, fast and non-invasive compared with gold standard.

Nerve fibres carrying signals from mechanoreceptors can be damaged due to a variety of causes, for example diabetes [18–20], carpal tunnel syndrome [11–12] or exposure to vibrating tools [21], and result in peripheral neuropathy. Following VPT changes over time would be useful as decreases can be an early indicator of nerve damage. This, however, triggers another area of interest; whether there are practice effects from repeated measurements and if these can affect the test results. When performing VPT testing in a repeated manner, little is known about the test-retest reliability. Available research in this area is contradicting, based on test-retest situations of different intervals or varying amounts of repeated tests [4, 7, 8, 16, 22–24]. To our knowledge, practice effects of multi-frequency vibrametry have not been studied in a population of healthy individuals. Hence, the aim of this study is to investigate practice effects in repeated measurements of VPTs using a multi-frequency test method.

## Methods

### Study design

This longitudinal study tested the vibrotactile sense of the pulp in the index and little finger of the right hand, in twenty healthy individuals on eight separate occasions. Data was collected during the period December 2016 to October 2018 by research nurses at the Department of Hand Surgery at Malmö University Hospital. VPTs were tested at seven frequencies (8, 16, 32, 64, 125, 250 and 500 Hz) using a vibrameter from VibroSense Dynamics AB.

### Subjects

Twenty healthy adults aged 26 to 65 years (mean 46.0 ± 11.1; 10 male, 10 female; 19 right- and 1 left-handed) were asked to participate in the study. Exclusion criteria were age below 18 or above 70, any neuropathic disease or conditions associated with peripheral neuropathy including spinal cord or spinal nerve root disorder, excessive use of alcohol or nicotine as well as no previous or present symptoms or treatment of carpal tunnel syndrome. Subjects were examined on eight different occasions: the first six with intervals of approximately one month between each test (mean 33 ± 6 days), referred to as time 0 and month 1–5, as well as one test at month 12 and one at month 18. Two subjects, one male and one female, did not participate in the last two examinations (participation denial *n* = 1, moved away *n* = 1).

### Ethics statement

The local ethics committee at Lund University approved the study (386/2007) of VPTs in healthy individuals and patients with diabetes mellitus. This study was conducted in accordance with the Helsinki declaration. Written informed consent was obtained from the subjects before starting the test.

### Multi-frequency vibrametry test situation

All vibration perception tests were conducted using a standard VibroSense Meter device and according to ISO 13091–1, Method A; without a surround and with a contact force of 0.15 ± 0.09 Newton between the finger and the probe, corresponding to a static skin indentation of approximately 1.5 mm [25]. The vibration probe had a diameter of 4 millimetres. Prior to the first test, participants received verbal instructions from the examiners regarding the test setup and they were encouraged to keep a high level of concentration. Room temperature was kept at a constant level of 20–22˚C and ambient noise or distractions were kept to a minimum in all tests. Subjects were seated comfortably with the right arm resting on a table in level with the probe. No visual contact with the probe was possible. Subjects were presented with a response button and received instructions as when to press and when to release. Measurements were performed on the pulp of the right index and little fingers, respectively. The finger pulp was placed on the probe and subjects were instructed to let it rest comfortably during the whole test sequence. Skin temperature was measured before starting and contact force to the probe was measured throughout the test to ensure that these were within limits defined in the ISO 13091–1 standard. A single vibration perception threshold test at 16 Hz was performed as a test run, but result from this initial test was not recorded. VPTs were then measured at seven frequencies, in a consecutive sequence from low to high (8, 16, 32, 64, 125, 250 and 500 Hz). All tests were automated and were running from start to finish without any interception from examiner or subject. For each frequency, vibration perception thresholds (VPTs) and vibration disappearance thresholds (VDTs) were registered in three cycles and graphically illustrated in a tactilogram. Perception is measured in dB, where decreasing dB values equals lowering of the VPT. Lowered thresholds throughout time indicates an improved perception. A detailed description of the VibroSense Meter and test protocol has been presented previously [6]. From beginning to end, the test lasted for approximately 8 minutes.

### Test-retest at eight different test occasions

VPTs in all twenty subjects were investigated on six different occasions, with intervals of 20–50 days (mean 33 ± 6). Eighteen subjects were tested on two additional occasions: at month 12 and 18 (mean 191 ± 33 days in between). Initial instructions as described above were given to all subjects on the first test occasion and in the following only if necessary. The same test equipment (standard VibroSense Meter) and test protocol was followed on all eight occasions. All tests were conducted in the same building, by the same three examiners and during daytime on a working day, except for one participant who ran all eight tests in the evening of a working day. In order to limit the effect of adaptation, subjects were not allowed to practice or use the vibrameter and when at test occasion, subjects were only allowed to run the frequency sequence once. In all test occasions, for all 20 subjects, every test resulted in three VPTs per frequency. Consequently, a total of 6547 VPT values were collected.

### Statistical analysis

Linear mixed model analyses were performed to investigate within-person differences over subsequent test occasions. The model was chosen as it includes systematic effects, such as practice effects, and allows test data from all eight test occasions to be included [26–27]. Analyses were performed on VPTs of each finger and frequency, separately. Fixed-effects part contained test occasions as an ordinal variable, and random-effects part contained the subjects. Autoregressive repeated covariance type was chosen as we assumed that eventual practice effects would follow over all test occasions, i.e. if any practice effects existed. Two separate analyses were performed; one analysis for the first six test occasions (time 0 to month 5) and one for the tests at time 0, month 12 and month 18. This approach was chosen in order to evaluate practice effects both when testing VPTs with intervals of one month, as well as within examinations of longer intervals. P-values <0.05 were considered statistically significant. In addition to the linear mixed model analysis, pairwise comparisons with Bonferroni correction were performed to investigate where the potential practice effects occurred, if they occurred at all. The statistical analyses were done using IBM SPSS Statistics Version 24 for Mac (Statistical Package for the Social Sciences, SPSS Inc., Chicago, Il, USA).

## Results

### Test-retest reliability in measurements with interval of one month

**Fig 1:**
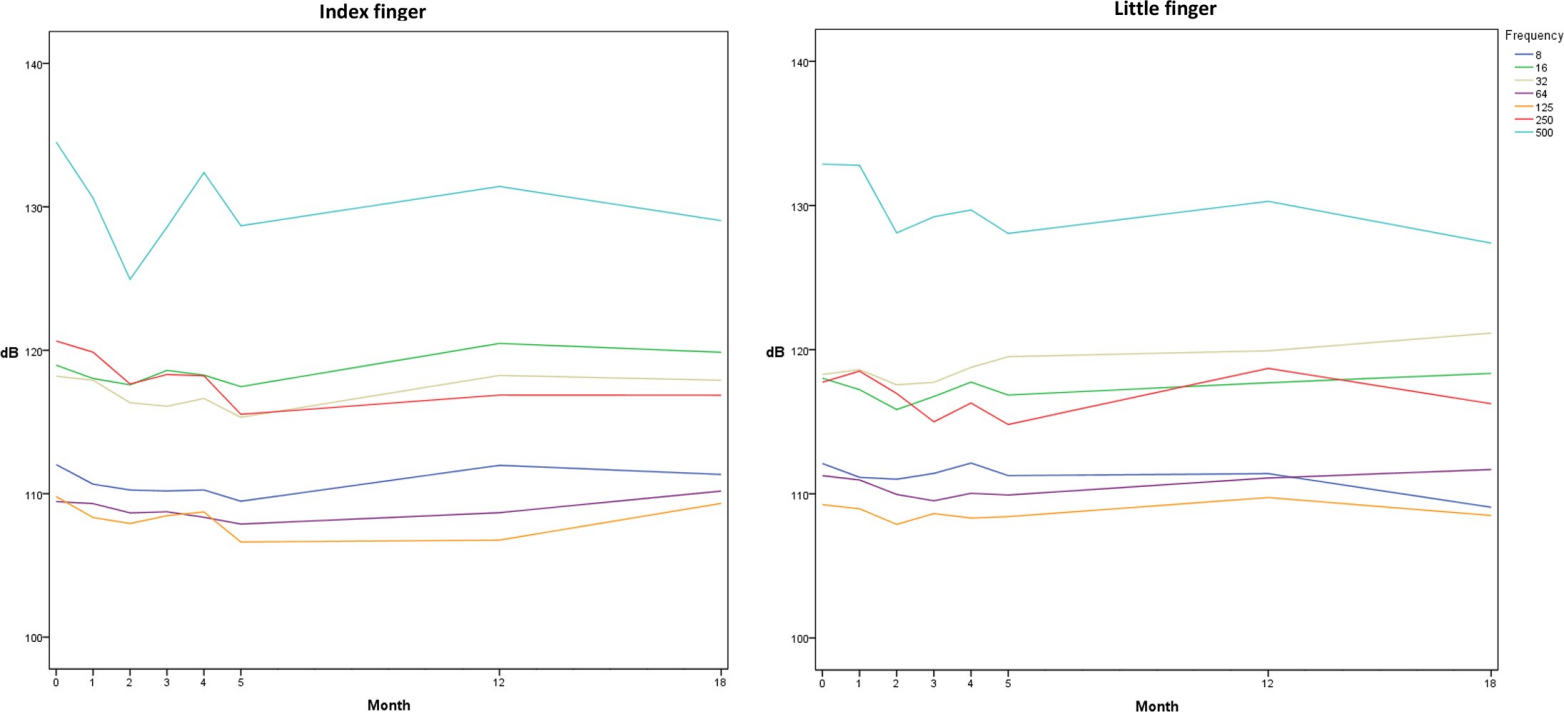
Vibration perception thresholds on eight test occasions. Mean values of vibration perception thresholds [dB] in 18 subjects for each finger, frequency and test occasion. Decrease in dB equals lowering of threshold, indicating an improved test result.

Linear mixed model analysis showed significant decreases in VPTs over the six month period in five out of seven frequencies tested in the index finger (8, 16, 32, 250 and 500 Hz), *p*<0.021. In the little finger, decreases were found at three frequencies (16, 250 and 500 Hz), *p*<0.020. Significant decreases were observed mainly on the test occasions of month 2 and 5 (Table 1). Mean values for each finger, frequency and test occasion are plotted in Fig 1.

**Table 1:** Mean values, mean differences and p-values for vibration perception thresholds in index and little fingers in tests from time 0 to month 5.

*Mean VPT*		*Mean difference*					*Mean VPT*	*p-value*
*Test occasion*	*Time 0*	*Month 1*	*Month 2*	*Month 3*	*Month 4*	*Month 5*	*Month 5*	
**Frequency [Hz]**								
***Index finger***								
8	112.194	-1.503	**-2.125**	-1.697	-1.943	**-3.149**	109.045	**0.010**
16	119.324	-1.100	-1.651	-0.457	-0.943	-2.801	116.623	**0.021**
32	118.988	0.522	-1.743	-3.325	-2.875	**-5.088**	113.900	**0.004**
64	109.926	-0.205	-1.049	-1.450	-2.226	-2.502	107.425	0.481
125	109.814	-1.057	-1.371	-1.463	-1.405	-3.326	106.488	0.120
250	122.776	-1.463	**-5.256**	**-5.690**	**-5.963**	**-9.019**	119.911	**0.000**
500	136.339	-3.394	**-8.148**	**-7.686**	-5.556	**-9.801**	126.538	**0.001**
***Little finger***								
8	111.467	-0.211	-0.698	0.067	0.799	0.618	112.085	0.635
16	119.266	-1.304	**-3.031**	-2.690	-2.470	**-3.743**	115.523	**0.020**
32	118.885	0.394	-0.556	-0.354	-0.699	-0.522	118.363	0.989
64	111.997	-0.830	-2.172	-2.520	-2.587	-2.984	109.013	0.264
125	110.148	-1.218	**-2.647**	-2.082	-2.374	-2.819	107.329	0.074
250	120.195	-0.194	**-3.837**	**-6.424**	**-4.730**	**-7.443**	112.752	**0.000**
500	134.372	-1.185	**-6.732**	-5.889	-5.701	**-7.843**	126.529	**0.003**

Pairwise comparisons of vibration perception thresholds in index and little fingers based on 20 subjects. Mean VPT values and mean differences (month X-time 0) in dB and p values for each frequency, highlighted in bold if significant. Lowering of dB indicates improved vibration perception threshold.

### Test-retest reliability within increased test intervals

Linear mixed model analyses were performed using data from the eighteen subjects participating in the tests of time 0, month 12 and month 18. Intervals between test occasions were 321–464 days (mean 381 ± 36) for time 0 and month 12, as well as 144–281 days (mean 191 ± 33) for month 12 and 18. Significant decreases in VPTs were found at three frequencies in the index finger (125, 250 and 500 Hz) and at one frequency in the little finger (250 Hz) (Table 2). Mean values for all test occasions, based on the 18 subjects that participated in all eight tests, are plotted in Fig 1.

**Table 2:** Mean values, mean differences and p-values for vibration perception thresholds in index and little fingers in tests at time 0, month 12 and month 18.

	*Mean VPT*	*Mean difference*		*Mean VPT*	*p-value*
*Test occasion*	*Time 0*	*Month 12*	*Month 18*	*Month 18*	
**Frequency *[Hz]***					
***Index finger***					
8	111.399	0.432	0.032	111.431	0.636
16	118.687	1.523	1.062	119.749	0.058
32	117.636	0.294	0.612	118.248	0.884
64	109.687	-1.047	0.294	109.981	0.075
125	110.145	**-3.074**	-0.816	109.328	**0.000**
250	120.323	**-3.576**	**-4.139**	116.184	**0.001**
500	134.066	-1.894	**-5.158**	128.908	**0.038**
***Little finger***					
8	111.159	0.536	-1.251	109.909	0.218
16	118.170	-0.603	-0.099	118.072	0.621
32	117.915	2.377	3.383	121.298	0.266
64	111.301	-0.479	-0.201	111.100	0.807
125	109.731	-0.732	-2.168	107.563	0.068
250	117.937	0.503	-2.268	115.669	**0.009**
500	131.719	-1.974	-4.738	126.982	0.090

Pairwise comparisons of vibration perception thresholds in index and little fingers based on 18 subjects. Mean VPT values and mean differences in dB (month X-time 0) and p values for each frequency, highlighted in bold if significant. Lowering of dB indicates improved vibration perception threshold.

### VPT changes in relation to age and gender

**Fig 2:**
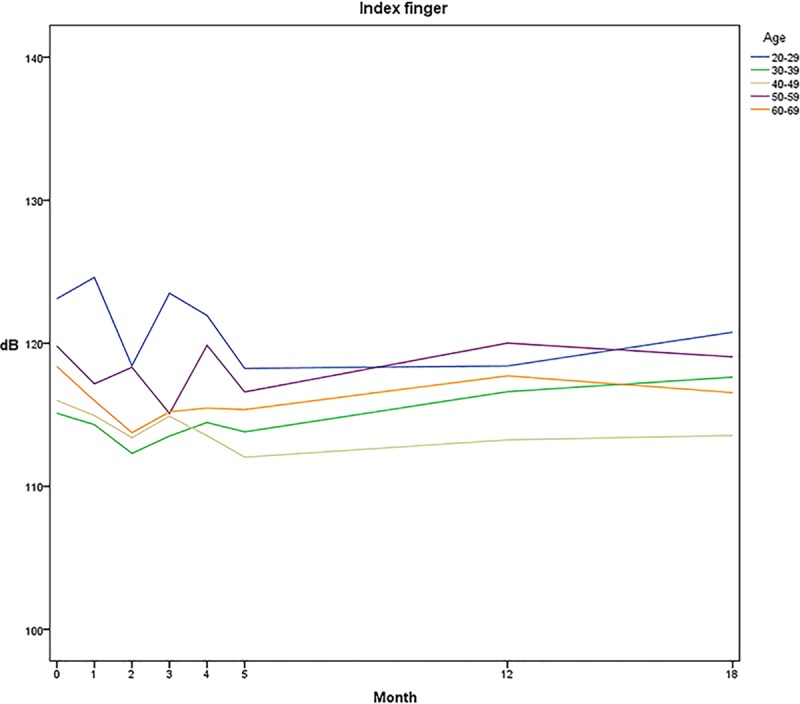
Vibration perception thresholds in age categories. Mean values of vibration perception thresholds [dB] in 18 subjects for five different age categories of ten years. Decreases in dB equals lowering of threshold, indicating an improved test result.

**Fig 3:**
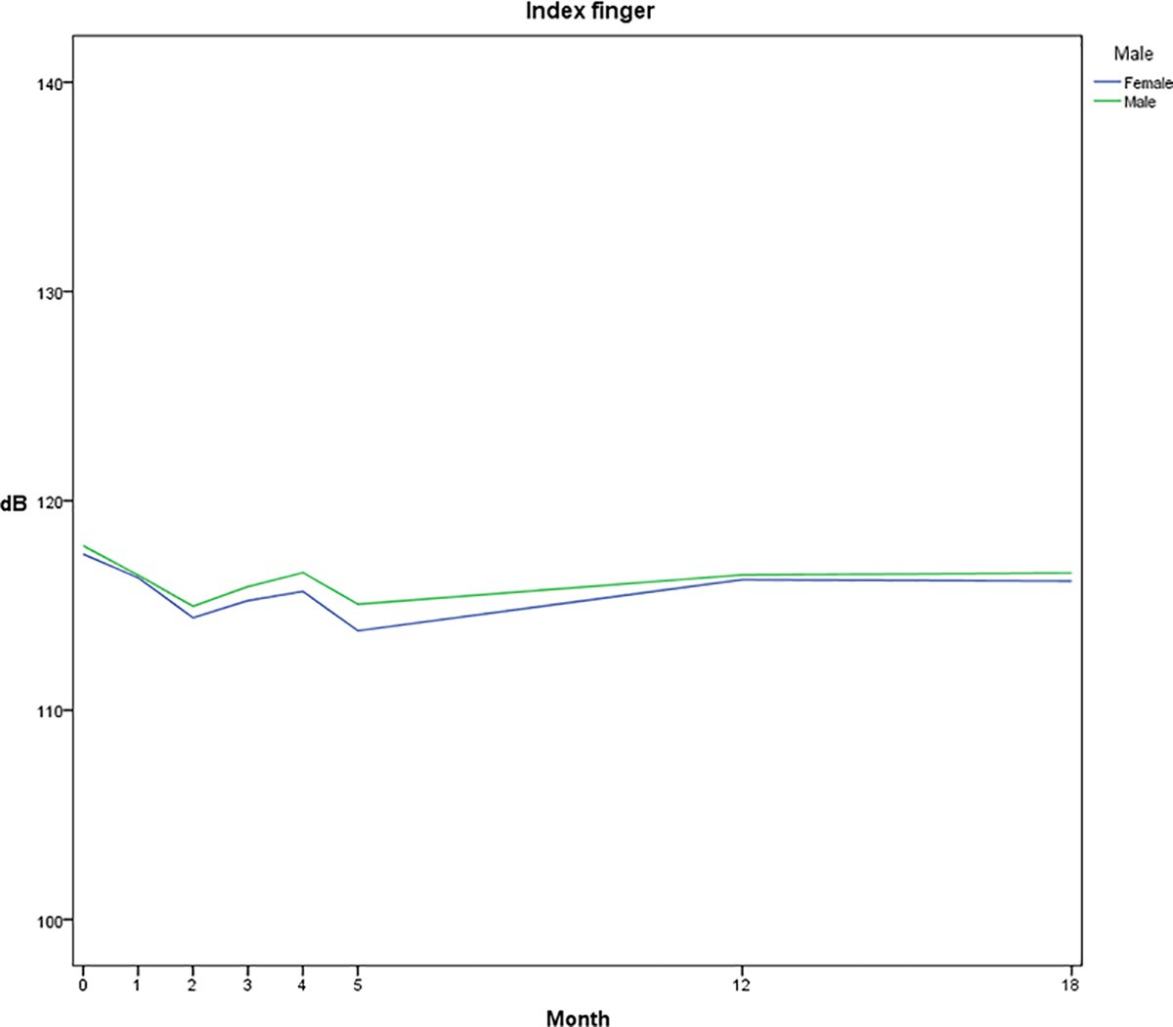
Vibration perception thresholds based on gender. Mean values of vibration perception thresholds [dB] in 18 subjects for men and women, separately. Decreases in dB equals lowering of threshold, indicating an improved test result.

Mean values of all subjects for all tests were divided in to five age groups, with intervals of ten years. Test results are distributed between values of 110 and 125 dB, with the youngest group (20–29 years) observed with the highest average thresholds. Participants between 40–49 years presented with the lowest thresholds on the eighth test occasion (Fig 2). Data of VPTs for men and women, respectively, showed that women are presented with slightly lower thresholds than men between the tests of time 0 and month 18 (Fig 3).

## Discussion

We found VPTs to be decreasing in five out of seven frequencies for the index finger, and three out of seven frequencies for the little finger, when examining with short intervals of one month, for a total period of six months. A decrease over time, when examining in a repeated manner, indicates an improved perception threshold and thereby a potential practice effect. However, changes in VPTs (Table 1) are ranging between 2 and 10 dB and not steadily downwards. Changes of 2–5 dB are considerably small and clinical importance is doubtful. Thus, changes could not be interpreted with certainty as a practice effect, rather than a measurement error. The mean differences of higher values, between 8 and 10 dB, are only observed at higher frequencies (250 and 500 Hz). Although, both intra- and inter-subject differences are generally larger and more widespread in the higher frequencies than in the lower ones, for example at 8 Hz. This reflects the difficulty of detecting higher frequencies, which were noticed and discussed with a majority of the subjects after performing the tests. Accordingly, we do not believe it is possible to draw any conclusions regarding practice effects with VPT changes of only 10 dBs.

The two additional tests, at month 12 and 18, were performed to evaluate the test-retest reliability over a time period of higher clinical relevance. Patients with diabetes, or other disease related to neuropathy, are probably to be examined with a minimum time-interval of six months. Thus, we compared results from month 12 and 18 with the test result at time 0. The sixth occasion, month 5, could not be used during these analyses due to the intensive practice and difficulties in interpreting results, regarding the previous period. However, between the tests of time 0 and month 12, subjects have been kept away from practicing for six months. Hence, we argue that this time period of twelve months reflects a six months rest. Changes seen from time 0 to month 5 are diminished and almost extinct when analyzing time 0 with month 12 and 18, exclusively. Maximum decrease observed was 5.2 dBs. As mentioned, the higher frequencies of 250 and 500 Hz seem to be more difficult to perceive and thus yield a diverging result, both within and among subjects. This wide-spread pattern could be causing the small but significant VPT changes.

In order to study changes in vibration perception over time, linear mixed model analyses were applied. The main limitations of using this method is the small population of only 20 subjects. Ideally, the size of the population should be somewhat larger in order to control for confounding effects. However, the proposed model was chosen since data from all subjects, frequencies and test occasions could be included and necessary adjustments were applied to exclusively investigate the within-person changes. In addition, pairwise comparisons were performed to verify the results from previous analyses and to detect the time point of potential practice effects. Values of VDTs were not included in the statistical analyses since the aim of the study was to investigate potential practice effects in vibration perception values, i.e. only VPTs were used. Visualization of VPTs (Fig 1) shows the various levels of vibration perception at different frequencies, which is in agreement with previous studies [6, 28]. General levels of VPTs are also in agreement with a currently ongoing study of the normative VPT values in an extensive number of subjects of different age, length, weight and gender (Ekman et al; to be published). Moreover, gender differences supports the notion that the intraepidermal nerve fiber density of the hand is lower in men than in women [29].

Even if the observed changes might be considered negligible, the question arises as to whether declines occur. Studying healthy individuals, where vibrotactile senses are expected to remain at the same level, any changes in test results would be considered to be due practice. Thus, significant decreases in VPTs could be derived from either learning the method, i.e. adaptation, or an actual improved vibration perception. The latter is referred to as practice effects and would yield a decline in VPT along with the intensity of training and exposure to the method. Adaptation, on the other hand, would lead to a reduced variability over time, resulting in a stable and equivalent result independent of further exposure. Adaptation could for example involve increased knowledge of the situation as well as timing and rhythm of the response to a perceived vibration. In order to detect pure practice effects, adaptation must be avoided. In this multi-frequency vibrametry test, VPTs are tested in a consecutive sequence from low to high frequencies. Preferably, frequencies would be tested in a random sequence within each test to completely avoid adaptation. However, the effect of adaptation was limited by the subjects not being allowed to practice in between tests, and by the fact that frequencies only were measured once.

Further influencing factors on the varying results are subject’s ability to concentrate and stay focused during the test. The way the probe is related to the pulp of the finger might also cause intra-subject variability [30]. These factors, together with the time of year (i.e. season) and body temperature, could be targets for future research. Although care was taken to ensure that all tests in this study were performed in an identical manner, results were not identical. Identifying factors influencing the intra-subject variability would favour a method adjusted for confounders. Such a method would detect even a minor change in vibration perception, thus enabling detection of early pathological signs. Although, if a patient displays with high consistency during several tests, emerging differences must be taken into bigger consideration, even though they are small. It is therefore important to study and consider the previous results of each patient individually when interpreting their results. Future research requires a larger population of healthy individuals to study the eventual adaptation and practice effects more thoroughly. Also, patients with pre-existing neuropathy constitute an interesting area to study since their potential practice effects might be reduced, thus allowing us to follow the actual decreases in perception.

## Conclusion

As the effect of continuous training is relatively small, and diminishing when increasing test intervals, we suggest that no adjustments are made regarding test method or interpretation of results when examining vibration perception thresholds in healthy adults.
